# Supplementary material for: Polyketide synthase gene clusters in the ericoid mycorrhizal fungus Oidiodendron maius: genome-wide prediction, expression profiling, and functional characterization of knockout mutants
Source: Front Plant Sci. 2026 Sep 16;17:1924430. doi: 10.3389/fpls.2026.1924430 (PMC13623903; doi:10.3389/fpls.2026.1924430)
Supplement: Supplementary file 1 [file SupplementaryFile1.zip › Supplementary Materials/Supplementary Folder 1.zip!/Oidma1_AssemblyScaffolds_Repeatmasked/knownclusterblast/cluster41/jgi.p_Oidma1_133633_mibig_hits.html]

| MIBiG Protein | Description | MIBiG Cluster | MiBiG Product | % ID | % Coverage | BLAST Score | E-value |
| --- | --- | --- | --- | --- | --- | --- | --- |
| AAK11531.1 | PaxG | BGC0001082 | Paxilline\_biosynthetic\_gene\_cluster | 38 | 57.063197026 | 212 | 1e-54 |
| ctg1\_orf003 |  | BGC0000688 | Copalyl\_diphosphate\_biosynthetic\_gene\_cluster | 38 | 57.6208178439 | 198 | 2e-50 |
| AAW88510.1 | geranylgeranyl\_diphosphate\_synthase | BGC0001081 | Lolitrem\_biosynthetic\_gene\_cluster | 38 | 54.0892193309 | 192 | 1.4e-48 |
| AAT65717.1 | geranylgeranyl\_diphosphate\_synthase | BGC0000629 | Aflatrem\_biosynthetic\_gene\_cluster | 38 | 51.4869888476 | 191 | 1.8e-48 |
| CAA75568.1 | geranylgeranyl\_diphosphate\_synthase | BGC0001270\_c3 | Gibberellin\_A3\_/\_gibberellin\_A1\_/\_gibberellin\_A7\_/\_gibberellin\_A4\_/ | 44 | 39.219330855 | 185 | 1.7e-46 |
| CAP07654.1 | geranylgeranyldiphosphate\_synthase | BGC0001269\_c2 | Gibberellin\_A3\_/\_gibberellin\_A1\_/\_gibberellin\_A7\_/\_gibberellin\_A4\_/ | 37 | 55.0185873606 | 183 | 6.6e-46 |
| BAD29970.1 | geranylgeranyldiphosphate\_synthase | BGC0000676 | Aphidicolin\_biosynthetic\_gene\_cluster | 34 | 53.717472119 | 173 | 8.9e-43 |
| CBF85177.1 | conserved\_hypothetical\_protein | BGC0000673 | Ent-pimara-8(14),15-diene\_biosynthetic\_gene\_cluster | 33 | 54.6468401487 | 171 | 2e-42 |
| ABF20220.1 | multi-functional\_prenyl\_transferase | BGC0001081\_c2 | Lolitrem\_biosynthetic\_gene\_cluster | 28 | 48.3271375465 | 84 | 3.2e-16 |
| AAT65718.1 | prenyl\_transferase | BGC0000629 | Aflatrem\_biosynthetic\_gene\_cluster | 30 | 35.3159851301 | 81 | 4.6e-15 |
| BAM84046.1 | prenyl\_transferase | BGC0001260 | Terpendole\_E\_biosynthetic\_gene\_cluster | 27 | 42.0074349442 | 80 | 6e-15 |
